# Our Hospitals and the National Relief Fund

**Published:** 1916-09-09

**Authors:** 


### OUR HOSPITALS AND THE NATIONAL RELIEF FUND.

### MORE FACTS AND FIGURES.*

A LARGE number of letters have been received from London and provincial hospitals in response to the invitation to supply particulars of the waiting lists of persons applying for in-treatment, and to estimate the income for the current year as compared with the known figures for 1914 and 1915. The information, it will be remembered, was sought in order that some idea might be formed as to the effect upon civilian applicants of the allocation of a large number of beds to military cases, and to discover if there is a case, arising out of the effects of the war upon institutional finance, upon which to base a claim upon the National Relief Fund, the object of which was originally stated to be the relief of all hardship caused by the abnormal economic conditions brought about by the war. We select from a large volume of correspondence some interesting facts, figures, and opinions bearing upon the points in respect of which information was sought. In examining this data, it must be remembered that effects as to pressure on in-patient accommodation are often secondary, and that the hospital with no military beds may easily be affected in this respect by the lack of civilian beds elsewhere.

### Conditions in London.

*St. George's Hospital.*—There are 75 male and 91 female applicants on the waiting list for in-patient treatment. The estimated income from subscriptions and donations for the current year is £6,000, as compared with £7,400 in 1915 and £6,300 in 1914.

*King's College Hospital.*—£60,000 owing to bankers and £5,650 to tradesmen. Position aggravated by large increases in prices.

*German Hospital.*—The committee having added 30 beds, there is no waiting list. The probable ordinary income is £11,000, as compared with £9,428 in 1915 and £12,960 in 1914.

*City of London Hospital for Diseases of the Chest.*—As is usual at this season of the year, little time elapses between the application of a suitable case and admission; but greater pressure is expected in the winter, owing to the present diminution in the treatment which is afforded by the Insurance Committees and Public Health Authorities.

*Hampstead General and North-West London Hospital.*—Twenty-six on waiting list. A deficit of £5,000 expected this year.

*Great Northern Central Hospital.*—There are 29 men, 66 women, and 35 children awaiting admission. The charity administers a number of beds for soldiers in an adjoining public library, in addition to those provided in the hospital.

*West Ham and Eastern General Hospital.*—"In the event of it being decided to make grants from the National Relief Fund, we consider that we have a very strong case for receiving a share." The 110 beds and cots are claimed to be inadequate to cope with the needs of a borough with 300,000 inhabitants. "There is, of course, a long list of applicants waiting in-patient treatment, as there always is, and always will be, so long as the above conditions prevail." There was a deficit last year of £3,108; the deficit at the present time is £6,500. Eighty beds have been set aside for soldiers, and twenty-three added to the original accommodation.

### Conditions in the Provinces.

*Manchester Children's Hospital* "has no grounds for making any claim on the National Relief Fund."

*Radcliffe Infirmary, Oxford.*—There are 61 patients waiting for vacant beds. There is no reason to anticipate any serious falling off in revenue from voluntary sources, but the chief difficulty is the enormous rise in cost.

*Manchester Royal Eye Hospital* "would be grateful for a grant from the National Relief Fund." There are 119 names on the waiting list. The income is normal, but the expenditure for 1916 is increasing rapidly.

*Cumberland Infirmary, Carlisle.*—30 beds in the infirmary and 25 in an adjacent building have been placed at the disposal of soldiers. There is a waiting list of 40 civilian names. The income has fallen short of the expenditure for three years.

*Royal Sussex County Hospital, Brighton.*—Waiting list 112. Ordinary income about normal.

*Royal Berkshire Hospital, Reading.*—The waiting list (63) is not so long as it often has been. The ordinary income shows no falling off, but the expenditure for this year is expected to exceed that of 1915 by £3,000.

*St. Bartholomew's Hospital, Rochester.*—Beds have been increased temporarily from 105 to 120, but there is a waiting list of 63.

*Ancoats Hospital.*—"We shall indeed be happy to receive a grant from the National Relief Fund, especially in view of the fact that we have admitted quite a number of women and children whose husbands and fathers are at the Front. We have had women in here whose income has not exceeded £1 or 25s., with three or four little children to look after; patients of this sort are quite unable to pay anything towards their treatment." There are 44 names on the waiting list.

*South Devon and East Cornwall Hospital, Plymouth.*—The committee, to cope with civilian applications, has found it necessary to reduce the number of military beds from 60 to 25, and the pressure has now been overcome and the list reduced to 7 women.

* Previous articles appeared August 12, 1916, p. 441, and August 19, 1916, p. 469.

*Huddersfield Royal Infirmary.*—Average waiting list during the last twelve months 50. The revenue is about normal. The board is decidedly of opinion that in any distribution of the National Relief Fund all voluntary hospitals throughout the kingdom should participate.

*Royal Infirmary, Gloucester.*—Waiting list 24. Estimated small increase in total revenue from voluntary sources owing to interest on investments.

*Infirmary and Dispensary, Bolton.*—Number of patients waiting for admission for the week ending August 5, 1916: Men, 58; women, 93; children, 256; total 407. Income 1914, £9,765; 1915, £11,263. There are 138 beds in the hospital.

*Royal Hospital, Sheffield.*—"We quite agree with the views expressed in your article, and certainly think that the voluntary hospitals, which have done an enormous amount of work of national importance, are entitled to a grant from the National Relief Fund." Sixty beds in the hospital are devoted to the use of the wounded. There are 181 civilian names on the waiting list. There were deficiencies in 1914 and 1915 of £5,970 and £6,247 respectively, and that for the present year is expected to be £6,000.

*Worcester General Infirmary.*—70 beds in the infirmary are occupied by soldiers, and there is a waiting list of 32 women and children. An excess of £1,250 of expenditure over income is expected in the current year's accounts.

*Bristol General Hospital.*—There are 7 men, 89 women, and 28 children waiting to be admitted to the civil wards. Deficits—1914, £5,794; 1915, £6,939; 1916 (estimated) at least £5,000.

*Royal Victoria Infirmary, Newcastle-on-Tyne.*—The number of applicants on the waiting list of this hospital for in-patient treatment is as follows: Males, 691; females, 585; children, 319; total, 1,595.

*Royal Southern Hospital, Liverpool.*—A small increase in income is expected to be shown by the accounts for 1915. Waiting list: Males, 73; females, 25; children, 50.

*Royal Sea-Bathing Hospital, Margate.*—The hope is expressed that if there is any distribution by the National Relief Fund institutions such as this shall participate. There are 113 names on the waiting list, and as the beds are occupied for long and indefinite periods, it will be months before the list will be cleared.

*Royal South Hants and Southampton Hospital.*—There are 48 patients waiting for beds. The receipts are expected to be approximately the same as in preceding years, but the expenditure will probably be £1,000 more.

*Royal Salop Infirmary, Shrewsbury.*—In order to meet the needs of the military authorities, and at the same time provide accommodation for the civil population, the number of beds has been increased from 120 to 150. The excess of expenditure over income has grown to £2,500 in consequence of the larger number of patients and the extra cost of supplies.

### The Quality of the Waiting List.

In view of the very alarming numbers on the waiting lists of some hospitals, notably at the Victoria Infirmary, Newcastle-on-Tyne, and Bolton Infirmary, the views of some of our correspondents as to the quality of the waiting list are interesting. One well-known secretary points out that a hospital waiting list is a combination of a dozen or more separate lists, each expressing the opinion of an individual, and that possibly a medical board, taking all the circumstances into consideration, might considerably reduce numbers. On the other hand, it is urged that at some institutions the list is not added to without careful consideration, and a number of cases for whom, in times of less pressure, admission would be desirable are treated as efficaciously as possible in the out-patient department.

